# Evaluation of Safety and Antileishmanial Efficacy of Amine Functionalized Carbon-Based Composite Nanoparticle Appended With Amphotericin B: An *in vitro* and Preclinical Study

**DOI:** 10.3389/fchem.2020.00510

**Published:** 2020-07-03

**Authors:** Mallikarjuna Rao Gedda, Prasoon Madhukar, Alok Kumar Vishwakarma, Vimal Verma, Anurag Kumar Kushwaha, Ganesh Yadagiri, Shyam Lal Mudavath, Om Prakash Singh, Onkar Nath Srivastava, Shyam Sundar

**Affiliations:** ^1^Infectious Disease Research Laboratory, Department of Medicine, Institute of Medical Sciences, Banaras Hindu University, Varanasi, India; ^2^Department of Physics, Institute of Science, Banaras Hindu University, Varanasi, India; ^3^Infectious Disease Biology Laboratory, Chemical Biology Unit, Institute of Nano Science and Technology, Habitat Centre, Mohali, India; ^4^Department of Biochemistry, Institute of Science, Banaras Hindu University, Varanasi, India

**Keywords:** visceral leishmaniasis, cytotoxicity, nanoparticles, graphene-CNT composite, antileishmanial activity

## Abstract

Visceral leishmaniasis (VL) has been a major health concern in the developing world, primarily affecting impoverished people. It is caused by a protozoan parasite *Leishmania donovani* and is characterized by immune dysfunction that can lead to deadly secondary infections. Several adverse side effects limit the existing treatment options; hence, the need of the hour is some drug option with high efficacy and no toxicity. To make targeted delivery of Amphotericin B (AmB), we have used amine-functionalized versions of carbon nanostructures, namely f-CNT and f-Graphene (f-Grap). The results with f-Grap-AmB, because of a much larger surface area, were expected to be better. However, the results obtained by us showed only marginal improvement (IC50 f-Grap-AmB; 0.0038 ± 0.00119 μg/mL). This is, in all likelihood, due to the agglomeration effect of f-Grap-AmB, which is invariably obtained with graphene. To resolve this issue, we have synthesized a graphene-CNT composite (graphene 70% and CNT 30% by weight). Because CNT is dispersed in between graphene sheets, the agglomeration effect is avoided, and our study suggests that the f-Composite-AmB (f-Comp-AmB) showed no toxicity against the murine J774A.1 macrophage cell line and did not induce any hepatic or renal toxicity in Swiss albino mice. The f-Comp-AmB also showed a remarkable elevation in the *in vitro* and *in vivo* antileishmanial efficacy in comparison to AmB and f-CNT-AmB or f-Grap-AmB in J774A.1 and Golden Syrian hamsters, respectively. Additionally, we have also observed that the percentage suppression of parasite replication in the spleen of the hamster was significantly higher in the f-Comp-AmB (97.79 ± 0.2375) treated group in comparison with the AmB (85.66 ± 1.164) treated group of hamsters. To conclude, f-Comp-AmB could be a safe and reliable therapeutic option over the other carbon-based nanoparticles (NPs), i.e., f-CNT-AmB, f-Grap-AmB, and conventional AmB.

## Introduction

Visceral leishmaniasis (VL) is a fatal protozoan disease caused by the intramacrophagic amastigote form of the *Leishmania donovani* parasite and is transmitted through the bite of the sand fly vector belonging to the genus *Phlebotomus* (Tiwari et al., 2018; Gedda et al., 2019b). The current treatment options are improving, but their clinical promise is limited by adverse side effects, high cost, resistance, storage problems, and cumbersome mode of administration. The side effects include nephrotoxicity, myocarditis, severe vomiting/diarrhea, gastrointestinal and cardiac toxicity, etc. (Gedda et al., 2019b; Singh et al., 2019). Another significant problem with conventional treatment is the likelihood of disease relapse. For instance, following the conventional drug treatment, recurrence of VL or post-kala-azar dermal leishmaniasis (PKDL) can occur, in which patients show macular, papular, or nodular skin lesions often containing heavily parasitized macrophages (Singh et al., 2016). Although a single dose of liposomal amphotericin-B formulation (AmBisome) has minimal toxicity and enhanced efficacy when compared to AmB (Sundar et al., 2010), the high cost of the drug and its subsequent lack of access by the people most commonly affected (from lower economic strata in the endemic regions of the Indian and African subcontinent) has been a major concern (Sinha et al., 2010). Recently, new drugs, such as oxaboroles (DNDI-6148) and nitroimidazole (DNDI-0690) by DNDi, are in the pipeline for preclinical development against VL and CL for an optimized pharmacological profile. Still, their complete clinical trials may take several years (DNDi, 2017). Hence, there is an urgent need to move toward obtaining definitive data for making recommendations on new drug delivery systems as soon as possible. Such novel delivery systems could more accurately target the intramacrophage parasite for the amastigote clearance. Nanomedicine, a promising field for infectious diseases, has retained the hope for better VL drugs in the coming time. Many research groups have employed several NP-based AmB formulations against the infected macrophage both at the *in vitro* and *in vivo* levels for the treatment of experimental leishmaniasis (Khatik et al., 2014; Asthana et al., 2015; Shahnaz et al., 2017).

Graphene and its subordinates are presently at the cutting edge of almost every emerging field of science and design, including biomedical applications that involve therapeutic delivery, advancement in biosensors, bacteriostatic imaging, photothermal therapy, etc. (Hu et al., 2010; Kuila et al., 2011; Robinson et al., 2011; Akhavan et al., 2012; Yang et al., 2013). The entrancing physicochemical properties, ease of synthesis, and adaptable generation combined with characteristic biocompatibility and simple biofunctionalization make graphene an alluring nano scaffold for medicinal purposes, such as in drug delivery in preclinical and clinical conditions (Sanchez et al., 2011; Novoselov et al., 2012; Amrollahi-Sharifabadi et al., 2018). f-Grap has also appeared as a promising nanocarrier for the effective delivery of drugs due to its ability to cross cell membranes and move into the intracellular environment (Mudavath et al., 2014). Graphene has extraordinary application potential, and it enjoys zero band gaps, is inert to reaction, and has a multilayer formation due to graphene sheet π-π stacking. Additionally, π-π stacking in the graphene sheet surface (area ~2,630 m^2^/g) with delocalized p-electrons can be utilized for effective ultra-high loading of AmB.

Despite the extraordinary application potential, it cannot be ignored that hydrophobic graphene sheets (GS) tend to agglomerate due to Van Der Waals forces, which seems to be the main concern for drug delivery (Rodriguez-Perez et al., 2013). But this can be nullified by the amine functionalization of GS, which acts as a safer alternative to graphene oxide for biomedical applications (Singh et al., 2012). The f-CNTs are no less effective in transporting therapeutic molecules. f-CNTs can covalently link nanotubes and biomolecules, such as peptides, proteins, and nucleic acids, facilitating their easy access through the cell membranes (Karimi et al., 2015). It has also been found that f-CNT doesn't alter the viability of intracellular targets because of their uptake by lymphocytes and macrophages. Our research group has reached these conclusions after exploring a variety of amine-functionalized NPs (f-CNT and f-Grap) appended with AmB (f-CNT-AmB and f-Grap-AmB) against experimental leishmaniasis upon administration through intraperitoneal and oral routes (Prajapati et al., 2011a,b; Mudavath et al., 2014, 2016). With effective results of f-CNT-AmB and f-Grap-AmB as a drug delivery system, we have functionalized and characterized the graphene and CNT composite NP (f-Comp with 70% graphene and 30% CNTs) to prevent the agglomeration of graphene (Patel et al., 2018) for the effective loading of AmB (f-Comp-AmB). This process yielded better antileishmanial activity with relatively less toxicity than AmB, f-CNT-AmB, and f-Grap-AmB.

## Materials and Methods

### Synthesis, Functionalization, and Characterization of f-CNT-AmB, f-Grap-AmB, and f-Comp-AmB

#### Synthesis of GS

The well-known modified Hummer's method was used to synthesize the GS in which 3 g of highly purified graphite powder was slowly added into the solvent containing a mixture of H_2_SO_4_ and H_3_PO_4_ followed by its oxidation with 15 mg of KMnO_4_ powder and simultaneous magnetic stirring for 12 h at 50°C to properly mix the contents (Patel et al., 2018). Then, 3 ml of H_2_O_2_ was added drop-wise into the solution until a yellow-brown color appeared that removes the excess amount of KmNO_4_. The graphite oxide powder thus obtained was washed with dilute HCl until the pH of the solution became 7. The powder was dried at 80°C under a vacuum (10^−3^ torr) for 12 h, followed by the thermal exfoliation of graphite oxide powder (100 mg) at 1,050°C under an inert argon (Ar) atmosphere.

#### Synthesis of CNT

CNTs were synthesized by a spray-assisted chemical vapor deposition technique (Patel et al., 2018). The precursors used were benzene (C_6_H_6_) and ferrocene (C_10_H_10_Fe). An optimum quantity of ferrocene powder was dissolved into a high-purity benzene solution. The solution was sprayed through the nozzle of the pyrolysis set up with the help of Ar gas into the preheated quartz tube of diameter 1 cm. The hollow carbon cylinder, which consisted of aligned CNTs, was formed onto the inner surface of the quartz tube. The tube whose inner surface contained the deposited CNT configuration was soaked overnight in dilute hydrofluoric acid. The CNTs were collected in powder form. CNTs were stirred into the solutions containing a mixture of H_2_SO_4_ and HNO_3_ (3:1) for 12 h to remove the Fe content.

#### Synthesis of Composite

Graphene (35 mg) and acid-treated CNTs (15 mg) were dispersed into ethanol solution in different beakers. Both the solutions were sonicated for 3 h, followed by their mixing and followed by sonication for 3 h more to obtain a homogeneous dispersion. The solution so obtained was stirred for 12 h with the help of a magnetic stirrer. The resulting solution was dried at 60°C to obtain the GS-CNT composite material.

#### Functionalization of GS, CNT, and GS-CNT

For the functionalization of freshly synthesized GS, acid-treated CNTs, and GS-CNT composite, a solution of 0.1 M L-cysteine was prepared in double-distilled water (Talat et al., 2015). Then, 25 mg of GS was added into 250 mL of double distilled water and then sonicated at room temperature for an hour to obtain a homogeneous dispersion. A few drops of L-cysteine solution was added in the GS solution and sonicated for 30 min, followed by stirring for 3 h at room temperature. The solution was thoroughly washed with double-distilled water by centrifugation at 10,000 rpm to remove the unbound L-cysteine. This washing process was repeated five times to ensure the removal of all the unbound L-cysteine. The powder so obtained was dried in an oven at 60°C overnight. The CNTs and GS-CNT composite were subjected to similar treatment for their functionalization. The functionalization with the amine group bearing L-cysteine enables these carbon carriers to bind with the carboxylic group of AmB readily.

#### Attachment of AmB to f-GS, f-CNTs, and f-GS-CNT

To attach the drug, at first, 50 mg of amine-functionalized GS, CNTs, and GS-CNT were dispersed into 20 mL of double-distilled water in three different beakers. Then, a drug solution was prepared by dissolving 50 mg of AmB into 20 mL of dimethyl sulfoxide (DMSO) followed by sonication for half an hour. Last, 20 mL of drug solution was added drop-wise into three different solutions containing dispersed f-GS, f-CNTs, and f-GS-CNT composite. The obtained solutions were sonicated for 30 h at room temperature. The solution was thoroughly washed with double-distilled water under the centrifugation at 10,000 rpm for 10 min to remove the unbound drug. The drug-attached f-GS (AmB-f-GS), CNTs (AmB-f-CNTs), and Comp (AmB-f-GS-CNT) were collected in powdered form and dried in a hot air oven at 60°C overnight.

#### Material Characterizations

Structural analysis of the samples was carried out by powder X-ray diffraction (XRD) technique using a PANalytical X'Pert PRO diffractometer with a Cu Kα beam (λ = 1.5415Å) operated at 40 kV and 40 mA. The surface morphologies of the different samples were investigated by scanning electron microscopy (SEM) using an instrument of FEI quanta 200. In SEM analysis, all the powder samples were mounted on a separate metallic stub with the help of carbon paper. The microstructural studies were done by using transmission electron microscopy (FEI Technai-20 G2; acceleration voltage = 200 kV). Before the application of the electron beam, the powder sample was sonicated in ethanol and then the dispersed sample mounted on a copper grid holder. Attachment of the drug and functionalization was done by Perkin Elmer (Spectrum 100 FT-IR Spectrometer) instrument. First, we have recorded the FTIR spectra of the potassium bromide (KBr) pellet as a background. A small quantity of the prepared sample was mixed with KBr powder with the help of agate mortar and pestle. The mixture was pelletized with the help of hydraulic pressure. The prepared pellet was put into a metallic holder to record the spectrum.

### Biological Activity

#### Macrophage Cell Culture

Murine J774A.1 macrophage cell lines (derived from reticulosarcoma) were procured from the National Centre for Cell Sciences (NCCS) and were used as a cellular host. Cell lines were cultured in RPMI 1640 liquid medium containing 50 mg/L gentamicin and 10% heat-inactivated fetal bovine serum (Thermo Fisher Scientific) in CO_2_ incubator with 5% CO_2_ and 95% humidity. After the confluency of the J774.A1 macrophage cell lines, they were scrapped from the culture flask and transferred to 50-ml tubes. Tubes were centrifuged at 2,500 rpm for 10 min at 4°C and then washed with 1X PBS twice and suspended in 1 ml complete RPMI 1640 medium, and finally, the number of cells was counted using a hemocytometer.

#### Culture of Parasite

*Leishmania donovani* LEM 138 (MHOM/IN/00/DEVI) parasite was cultured *in vitro* in liquid medium (M199) supplemented with 10% heat-inactivated FBS and antibiotics in a BOD incubator. After the growth of the parasite, it was transferred to 50-ml tubes. Tubes were then centrifuged at 2,000 rpm for 10 min at 4°C and then washed with 1X PBS twice and suspended in 1 mL complete M199 medium, and the number of parasites was counted using a hemocytometer.

#### Animals

Male Swiss albino mice, *Mus musculus* (30–40 g), were purchased from the Central Animal Facility, Institute of Medical Sciences (IMS), Banaras Hindu University (BHU), Varanasi, India for *in vivo* toxicity assay and male Syrian golden hamsters, *Mesocricetus auratus* (50–60 g) were purchased from the Central Drug Research Institute (CDRI) animal house facility, Lucknow, India for *in vivo* antileishmanial activity. Both studies were performed using procedures accepted by the Central Animal Ethics Committee (CAEC), IMS, BHU (CAEC number Dean/2014/CAEC/615). The guidelines of the Council for the Purpose of Control and Supervision of Experiments on Animals (CPCSEA), Ministry of Environment and Forests, Government of India were strictly followed.

#### *In vitro* Assessment of Cytotoxicity

The different treatment groups, AmB, f-CNTs, f-CNT–AmB, f-Grap, f-Grap-AmB, f-Comp, and f-Comp-AmB, were assessed for their cytotoxic effects. For this purpose, nearly 5 × 10^4^ J774A.1 macrophages were aliquoted into 96-well plates and incubated in triplicate with AmB (0.005–0.64 μg/mL), f-CNTs (0.0625–8 μg/mL), f-CNT–AmB (0.005–0.64 μg/mL), f-Grap (0.0625–8 μg/mL), f-Grap–AmB (0.005–0.64 μg/mL), f-Comp (0.0625–8 μg/mL), or f-Comp-AmB (0.005–0.64 μg/mL) for 72 h at physiological temperature, 5% CO_2_, and analyzed using a 3-(4,5-dimethylthiazol-2-yl)-2-5-diphenyl tetrazolium bromide (MTT) assay. The untreated cells served as controls, and their optical density (OD) is taken as a measure of 100% survival. This experiment was performed twice to ensure the validity of the finding and reproducibility, and the concentration required to kill 50% of the cells (CC_50_) was calculated from the graph of ODs plotted against varying drug concentrations.

#### *In vitro* Antileishmanial Activity Against Intracellular Amastigotes

The J774A.1 macrophages were seeded on eight-chamber Lab Tek tissue culture slides (USA Scientific, Inc., Ocala, FL, USA) at a density of 5 × 10^4^ cells/well and allowed to adhere for 2 h inside a CO_2_ incubator with 5% CO_2_ at 37°C. The nonadherent macrophages were removed by washing the wells twice with serum-free RPMI 1640 medium. The adherent macrophages were then infected with the metacyclic stage of *L. donovani*, maintaining a *Leishmania*:macrophage ratio of 10:1 in a 200-μL final solution of a complete RPMI 1640 medium overnight. Then, 24 h postincubation, free promastigotes were washed with serum-free RPMI 1640 medium, and infected macrophages were incubated with AmB (0.04–0.00125 μg/mL), f-CNT (0.04–0.00125 μg/mL), f-CNT-AmB (0.04–0.00125 μg/mL), f-Grap (0.04–0.00125 μg/mL), f-Grap-AmB (0.04–0.00125 μg/mL), f-Comp (0.04–0.00125 μg/mL), and f-Comp-AmB (0.04–0.00125 μg/mL) and in duplicate for 72 h with 5% CO_2_ at 37°C except in the control well, followed by methanol fixing for a minute and staining with Giemsa (Qualigens, Mumbai, India). In each well, at least 100 macrophage nuclei were counted for estimating the percentage of infected macrophages, and the number of amastigotes per 100 macrophages and the IC_50_ (concentration of drug that inhibits 50% of *L. donovani* amastigotes) of each drug was calculated (Manandhar et al., 2008).

#### *In vivo* Toxicity Assay

The *in vivo* toxicity of AmB, f-CNT, f-CNT-AmB, f-Grap, f-Grap-AmB, f-Comp, and f-Comp-AmB was assessed in 84 Swiss albino mice (25 ± 5 weeks of age) with a 5-day course of daily intraperitoneal injection using 5, 10, and 20 mg/kg dose regimens in 12 mice with four mice for each concentration. The control groups, comprising four mice each, were injected with PBS. After the 5-day course, mice were euthanized, blood was drawn, and serum was separated by centrifugation. Serums were used to assess hepatic and renal function by quantifying the levels of biomarkers, such as aspartate transaminase (AST), alanine aminotransferase (ALT), urea, and creatinine to evaluate hepatotoxicity and nephrotoxicity, respectively, using commercially available kits (Autozyme GPT; Accurex Biomedical Pvt. Ltd., Mumbai, India. Enzopak SGOT; Reckon Diagnostics Pvt. Ltd., Gujarat, India. Lyphozyme Urea Berthelot; Beacon Diagnostics Pvt. Ltd., Gujarat, India. Autozyme creatinine; Accurex Biomedical Pvt. Ltd., Mumbai, India).

#### *In vivo* Antileishmanial Efficacy

Hamsters are an outstanding model of *L. donovani* infection as they share several common medical and immunopathological aspects with human VL and can endure regular spleen biopsies while being tested for the parasite burden. Eighty-eight male hamsters (4–6 weeks of age) were infected by intracardiac injection of 1 × 10^7^ promastigotes of *L. donovani* LEM138. Eight hamsters were randomly selected, collectively from all the groups, and sacrificed to isolate the spleen for confirmation of the infection by Giemsa staining of the splenic smears. There were seven treatment groups, each with three subgroups, along with one positive and one negative control, consisting of four animals each. AmB, f-CNT, f-CNT-AmB, f-Grap, f-Grap-AmB, f-Comp, and f-Comp-AmB were reconstituted for *in vivo* administration in 1X PBS at a concentration of 5, 10, and 20 mg/kg body weight. The first, second, and third subgroups were treated with 5, 10, and 20 mg/kg of AmB; the fourth, fifth, and sixth groups were provided with 5, 10, and 20 mg/kg of f-CNT; the seventh, eighth, and ninth groups with 5, 10, and 20 mg/kg of f-CNT-AmB; the 10th, 11th, and 12th groups with 5, 10, and 20 mg/kg of f-Grap; the 13th, 14^th^, and 15th groups with 5, 10, and 20 mg/kg of f-Grap-AmB; the 16th, 17^th^, and 18th groups with 5, 10, and 20 mg/kg off-Comp; and the 19th, 20th, and 21st groups were treated with 5, 10, and 20 mg/kg off-Comp-AmB intraperitoneally for five consecutive days while the control group received an equal volume of 1X PBS (vehicle). Autopsies were conducted on the seventh day, and the spleen was surgically removed for further examination. Postautopsy, the weight and length of the spleen were measured immediately, and dabbing was done to get imprints on the glass slides. The slides were then examined for the estimation of parasite burden, percentage of inhibition of parasite load, and percentage of suppression of parasite replication (Manandhar et al., 2008). This experiment was repeated for reproducibility.

#### Statistical Analysis

The CC_50_ and IC_50_ values were calculated by the latest version of GraphPad Prism software, and a one-way ANOVA test was applied to determine the cytotoxicity and antileishmanial activity with *p* < 0.05 as the significant value.

## Results

### Structural Studies

**Figure 1:**
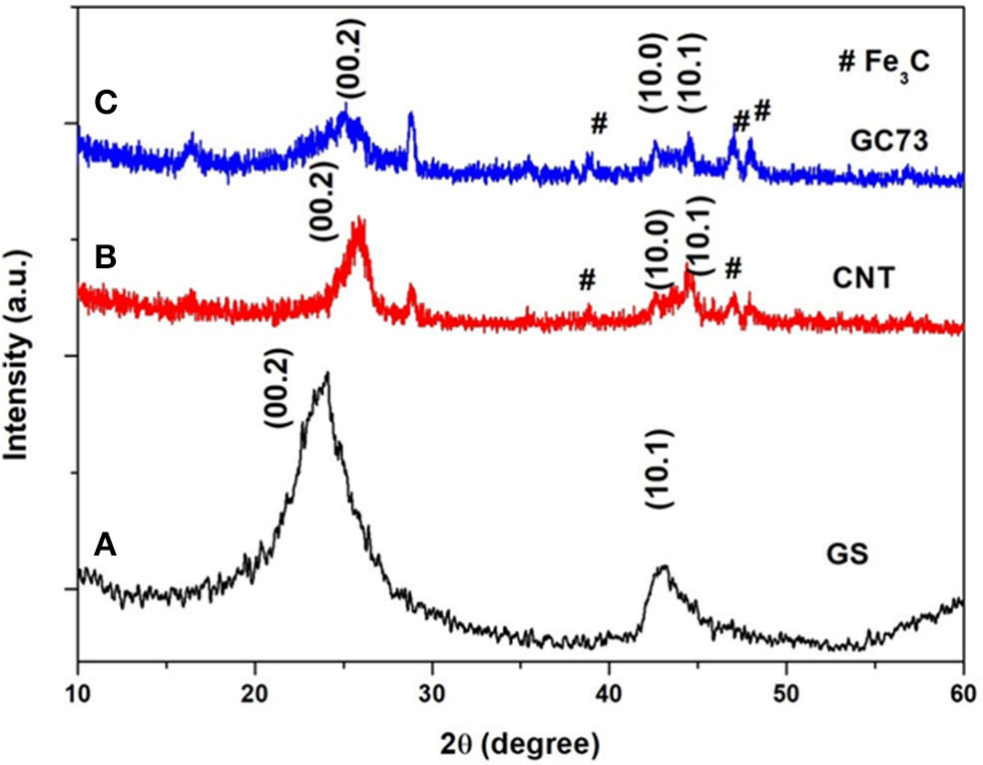
X-ray powder diffraction patterns of the **(A)** graphene sheet (GS) **(B)** carbon nanotube (CNT), and **(C)** composite (graphene-CNT). The composite with 70% graphene and 30% CNT has shown structural quality and stability with fewer chances for agglomeration.

To unravel the structural and microstructural details, the as-synthesized GS, CNT, and GC73 (GS-CNT composite) were characterized by the XRD technique, which is shown in Figure 1. Figure 1A shows typical XRD patterns of GS. The weak and broad peak corresponding to the plane (00.2) at 2θ value 23.86° indicates the formation of thermally exfoliated GS. Figure 1B shows the XRD patterns of the prepared randomly oriented CNTs. The peak corresponding to the plane (00.2), which is relatively sharp, indicates the formation of CNTs. XRD patterns of GS and CNT composite are shown in Figure 1C, which suggests that the peak corresponding to the plane (00.2) shifts toward the lower angle side (2θ = 24.995) and becomes broad, which is due to the combined size effects of GS and CNT.

### Morphological Studies of GS, CNT, and GS-CNT Composite

**Figure 2:**
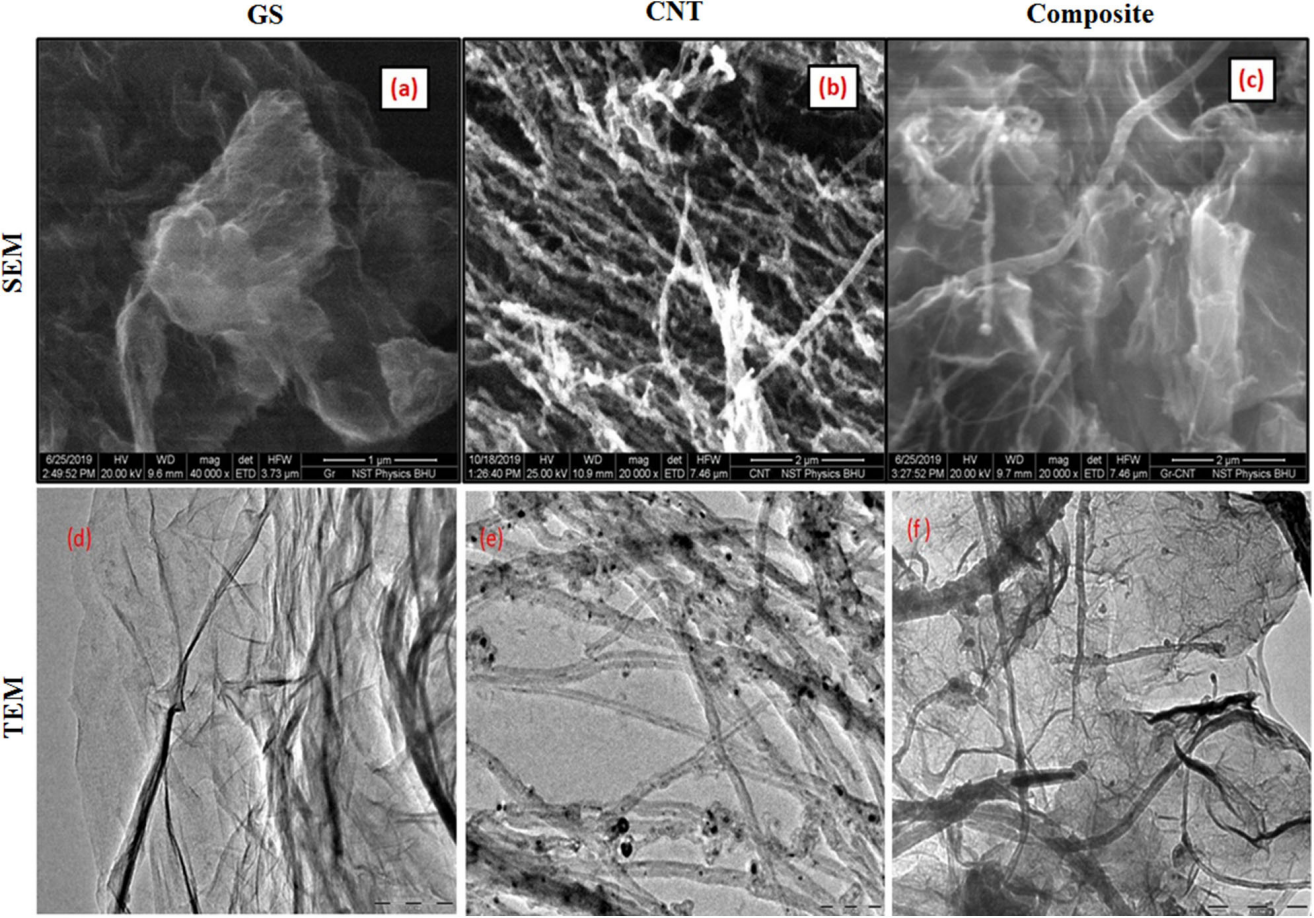
Scanning electron microscopy (SEM) shows the surface topology of the **(a)** graphene sheet (GS), **(b)** carbon nanotube (CNT), and **(c)** composite (graphene-CNT). The TEM image shows the presence of the **(d)** graphene sheet (GS), **(e)** carbon nanotube (CNT), and **(f)** composite (graphene-CNT).

The scanning electron microscopy (SEM) technique was employed to observe the surface morphology of the as-synthesized material. Figure 2 shows representative SEM micrographs of as-prepared materials. Figure 2a shows the planar sheets with wrinkles, which indicates the formation of GS. Figure 2b reveals the formation of randomly oriented CNTs. Figure 2c shows the surface morphology of the GS and CNT composite (GC73). It is clear from Figure 2c that the presence of CNT prevents the agglomeration of GS. It may be mentioned that, for CNT, the carbons are in mixed sp^2^ and sp^3^ states, and in graphene, the carbon is in purely the 2S state.

### Microstructural Studies of GS, CNT, and GS-CNT Composite

We have used the transmission electron microscopy (TEM) technique to investigate the microstructure of the as-prepared carbon nano variants (GS, CNT, and GS-CNT composite). Figure 2d exhibits the TEM image of GS. The transparent sheets with the presence of wrinkles indicate the formation of graphene. Figure 2e shows the TEM image of a randomly oriented carbon nanotube. Figure 2f represents the TEM image of the GS-CNT composite, which indicates the dispersion of CNTs inside the GS. The presence of CNTs inside the GS prevents the agglomeration of graphene.

### FTIR Studies of f-GS, f-CNT, and f-GS-CNT-AmB

**Figure 3:**
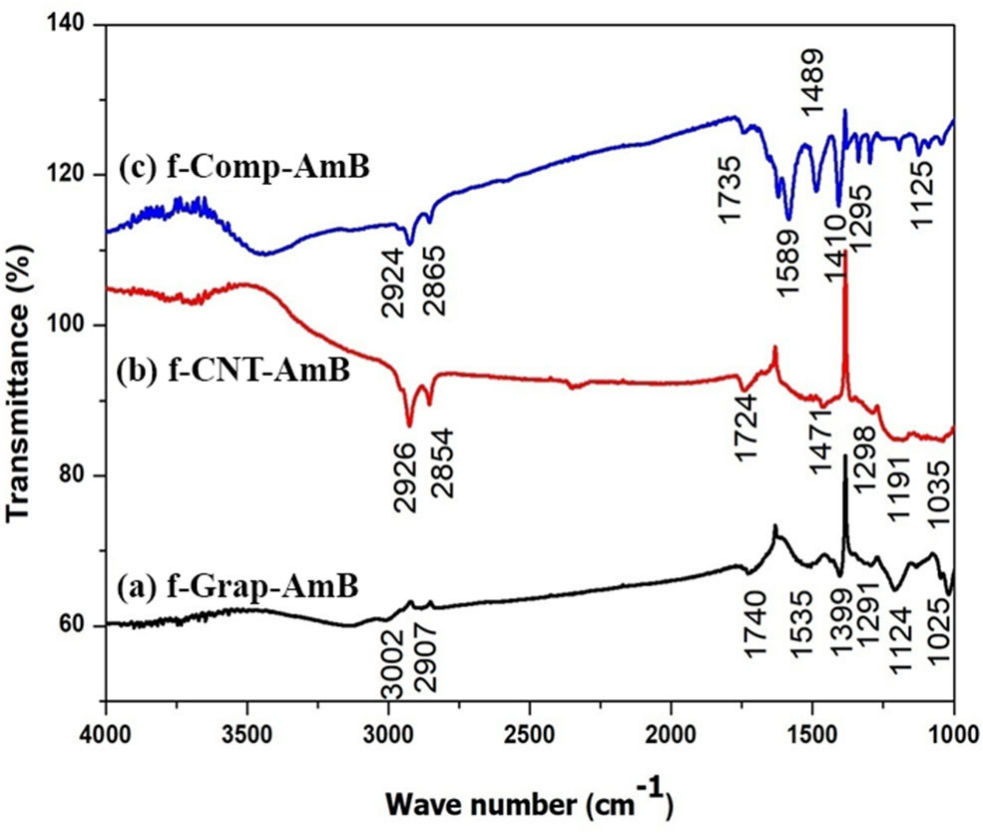
Fourier-transform infrared spectroscopy (FTIR) spectrum of **(A)** f-CNT-AmB, **(B)** f-Grap-AmB, and **(C)** f-Comp-AmB, which shows the data of characterization of functionalized NP with a peak at 1,637 cm^−1^ that represents the carbon skeleton and peaks at 2,930 and 3,492 cm^−1^ that reveal the C-H bond of CH_2_ and the N-H bond of NH_2_, confirming the amine attachment in CNTs, graphene, and composites. The peaks at 1,750 and 1,150 cm^−1^ represent C=O and C-O bonds of the ester group, and the peak at 1,050 cm^−1^ confirms the C-O-H bond present in AmB attached to f-CNTs, f-graphene, and f-composites.

To investigate the functionalization of carbon nano variants (f-GS, f-CNT, and f-GS-CNT) with AmB, we have performed FTIR measurements, which are shown in Figure 3. Figure 3A shows the FTIR spectrum of the drug attached with amine-functionalized GS. The peak corresponds to 3,003 cm^−1^, and 2,907 cm^−1^ is due to C-H stretching. The peak at 1,535 cm^−1^ is due to the stretching mode of the C=C, which is present in GS. The peak corresponding to 1,291 cm^−1^ is due to the stretching of C-N, which confirms the GS functionalized with an amine. The peak at 1,124 cm^−1^ is due to the presence of the C-O bond, and the peak corresponding to 1,740 cm^−1^ is due to the carbonyl C=O stretching, which is present in AmB. These observations confirm that the drug was attached with amine-functionalized GS. Figure 3B shows the FTIR of thre drug attached with amine-functionalized CNT. The peaks present at 2,926 and 2,854 cm^−1^ are due to C-H stretching. The peak at 1,471 cm^−1^ is due to the stretching mode of the C=C. The peak corresponding to 1,298 cm^−1^ is due to the stretching of C-N, which confirms the CNT functionalized with an amine. The peak at 1,191 cm^−1^ is due to the presence of the C-O bond. The peak corresponding to 1,724 cm^−1^ is due to the carbonyl C=O stretching, which is present in AmB, which confirms that the drug was attached with functionalized CNT. Figure 3C indicates the FTIR of the drug attached with amine-functionalized f-GS-CNT. The peaks at 2,924 and 2,865 cm^−1^ indicate the C-H stretching. The peak at 1,489 cm^−1^ is due to the stretching mode of the C=C. The peak corresponding to 1,295 cm^−1^ is due to the stretching of C-N, which confirms the f-GS-CNT functionalized with an amine. The peak at 1,295 cm^−1^ is due to the presence of the C-O bond. The peak corresponding to 1,735 cm^−1^ is due to the carbonyl C=O, which confirms that the drug was attached with f-GS-CNT. The quantity of loading of AmB for the carbon nano variants (f-GS, f-CNT, and f-GS-CNT) as checked from the FTIR peaks was found to be similar.

### *In vitro* Toxicity

Our present toxicity study on these NPs showed that CC_50_ of the compounds against the J774A.1 cell line were f-Grap–AmB (0.57 ± 0.125 μg/mL), f-Comp-AmB (0.604 ± 0.171 μg/mL), AmB (0.63 ± 0.157 μg/mL), f-CNT–AmB (0.63 ± 0.179 μg/mL), f-Comp (7.12 ± 0.897 μg/mL), f-Grap (7.84 ± 2.6 μg/mL), and f-CNTs (8 ± 1.05 μg/mL).

### *In vitro* Antileishmanial Activity

**Figure 4:**
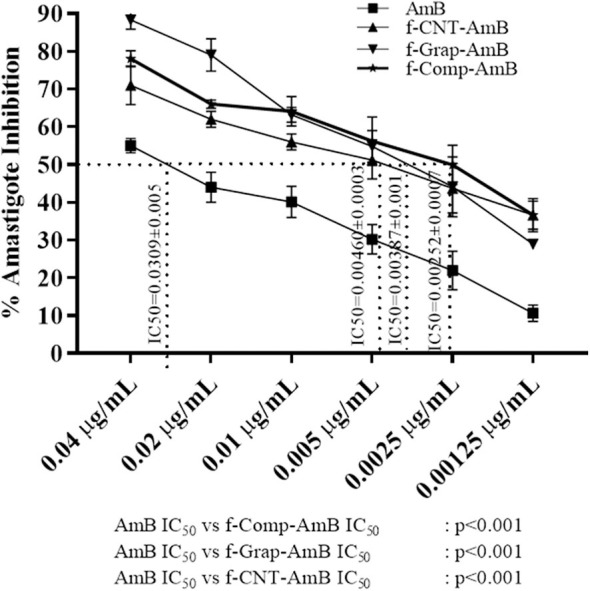
Percentage amastigote inhibition and the IC_50_ values of AmB, f-CNT–AmB, f-Grap–AmB, f-Composite–AmB. The results were analyzed using one-way ANOVA and expressed as Mean ± *SD* with *p* < 0.05 representing significance.

Amastigotes, the infective form of *Leishmania*, reside and replicate in the macrophages. Reduction in the number of intracellular amastigotes of infected macrophages is indicative of the better antileishmanial activity of a drug formulation. The antileishmanial activity of the compounds was AmB, IC_50_ = 0.0309 ± 0.00501 μg/mL; f-CNT-AmB, IC_50_ = 0.00460 ± 0.00038 μg/mL; f-Gr-AmB, IC_50_ = 0.00387 ± 0.00119 μg/mL; f-Comp-AmB, IC_50_ = 0.00252 ± 0.00078 μg/mL as shown in Figure 4. The potency of the drugs in inhibiting the intramacrophage parasites is 6.7, 7.98, and 12.22 times more than AmB for f-CNT-AmB, f-Gr-AmB, and f-Comp-AmB, respectively (*p* < 0.001). This gives substance to the conclusion that f-Comp-AmB is a better antileishmanial formulation compared to conventional AmB in comparison with the conventional f-CNT-AmB and f-Gr-AmB.

### f-Comp-AmB Did Not Induce Hepatorenal Toxicity in Swiss Albino Mice

Toxicity is one of the major concerns associated with drug formulations. So, we tried to find out the potential *in vivo* toxicity of the formulations in Swiss albino mice. There were no evident indications of toxic effects for AmB, f-CNT, f-CNT-AmB, f-Grap, f-Grap-AmB, f-Comp, and f-Comp-AmB injected mice at a dose of up to 20 mg/kg within 5 days. There was no significant difference in the body weights of the treatment group when compared with the control groups, and none of the groups showed mortality. The blood serum biochemistry of the renal and hepatic enzymes, urea (14.75–18.75 mg/dL), creatinine (0.3225–0.4850 mg/dL), AST (35.75–51 IU/L), or ALT (40.25–51.25 IU/L) showed no observable difference between the control and treatment groups at all doses, and all were within the normal reference range (Supplementary Table 1).

### f-Comp-AmB Showed Improved Antileishmanial Activity in the Hamster Model of VL

**Figure 5:**
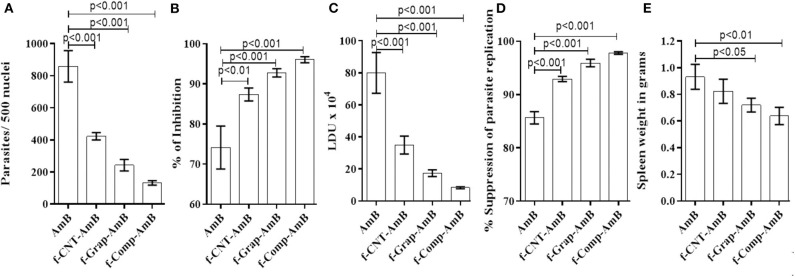
*In vivo* evaluation of the antileishmanial activity of drug formulations based on the **(A)** parasite count per 500 macrophage nuclei, **(B)** spleen weight **(C)** LDU, **(D)** percentage suppression of parasite replication, and **(E)** percentage inhibition of parasitic load correlated with hamsters of different experimental groups. The results were analyzed using one-way ANOVA and expressed as Mean ± *SD* with *p* < 0.05 representing significance.

The effects of both sets of *in vivo* studies were conflated to produce a mean value for evaluation. There was no significant difference in the weight of hamsters in the control and treatment groups. f-Comp-AmB showed remarkable improvement in the antileishmanial activity in comparison to AmB, which is evident from the side-by-side bar patterns of parasite count per 500 macrophage nuclei (Figure 5A and Supplementary Figure 1), Leishman Donovan units (LDU) (Figure 5C), percentage suppression of parasite replication (Figure 5D), and percentage inhibition of the parasite load (Figure 5B). These results are corroborated by the weight of the spleen in different treatment groups (Figure 5E). The percentage inhibition of parasites in the spleens of f-Comp-AmB treated *Leishmania* infected hamsters (96.04 ± 0.7526) was higher when compared with conventional AmB treated *Leishmania*-infected hamsters (74.13 ± 5.338) (*p* < 0.001, Supplementary Table 2). f-Comp-AmB also showed a more significant reduction in the parasite burden (8.351 ± 0.633 X 10^4^ LDU) than in AmB treated groups (79.97 ± 12.72LDU) (*p* < 0.001, Supplementary Table 2). Even the percentage suppression of parasite replication was higher for f-Comp-AmB (97.79 ± 0.2375) when compared with AmB-treated *Leishmania*-infected hamsters (85.66 ± 1.164) (*p* < 0.001 Supplementary Table 2). The percentage inhibition of parasites and percentage suppression of parasite replication by f-CNT-AmB and f-Grap-AmB were found to be 87.36 ± 1.613 (*p* < 0.01), 92.92 ± 0.4802 (*p* < 0.001) and 92.77 ± 1.039 (*p* < 0.001), 95.92 ± 0.7118 (*p* < 0.001), respectively, which are slightly higher than the conventional AmB treated groups while lower than that of f-comp-AmB. Above this, a significant reduction was observed in the spleen size of f-Comp-AmB-treated hamsters (3.167 ± 0.30 cm) in comparison with the infected hamsters (4.73 ± 0.40 cm) (Supplementary Figure 2) (*p* = 0.005), suggesting a decline in the parasite burden.

## Discussion

The present work explores the *in vitro* and *in vivo* toxicity and antileishmanial efficacy of a novel treatment approach for VL: amine-functionalized AmB attached composite NP (70% f-Grap and 30% f-CNT) against f-Grap attached AmB, f-CNT attached AmB, and solely conventional AmB. The amine functionalization enables the composite to attach with the carboxylic group of AmB, forming a stable f-Comp-AmB conjugate, and the bond being covalent prevents the dissociation of AmB during drug delivery. Other carbon nanomaterials have also been extensively explored for drug delivery (De Jong and Borm, 2008).

The f-Comp-AmB showed significantly enhanced antileishmanial activity against the intracellular amastigotes of *L. donovani* in the J774A.1 cell lines with 12.2-fold improvement in IC_50_ values in comparison with conventional AmB. Although the other functionalized carbon nanomaterials, i.e., f-Grap-AmB and f-CNT-AmB, have shown only 7.98- and 6.71-fold improvement in the *in vitro* antileishmanial activity over the conventional AmB, which has been in corroboration with our previous *in vitro* results (Prajapati et al., 2011a,b; Mudavath et al., 2014). The enhanced uptake of f-Comp-AmB by macrophages present superior *in vitro* antileishmanial efficacy than the other nanoformulations, i.e., f-Grap-AmB, and f-CNT-AmB, and hence, it can be a better drug-delivery system of AmB for the intramacrophagic amastigotes. These results are in agreement with the outcomes of an *in vivo* experiment involving hamster models of *L. donovani* infection. The f-Comp-AmB showed a remarkable decline in parasite load and enhanced intracellular delivery and effectiveness of AmB when administered to the infected hamsters in comparison to other alternatives, such as AmB, f-CNT-AmB, and f-Grap-AmB.

The toxicity and high cost of the AmB treatment in VL raise concerns that can be potentially addressed by amine functionalization of carbon-based NPs although, in recent years, several safer formulations of AmB (AmBisome, Fungizone, Amphotec, and Abelcet) have been made commercialized at a significantly increased rate. The use of carbon-based NPs for AmB drug delivery is considerably cost-effective (Sanchez et al., 2011) although cytotoxicity has played a substantial role in limiting several harnessed NPs for the drug-delivery applications. Also, both CNT and graphene oxide have shown serious concerns due to their cytotoxicity and absence of hemocompatibility (Singh et al., 2011; Gedda et al., 2019a). But the amine functionalization of carbon-based NPs, i.e., CNT and graphene, was shown to be a safe alternative (Prajapati et al., 2011a; Mudavath et al., 2014), and hence, this study has utilized their amine-functionalized composite variant. The IC_50_ values are much lower than CC_50_ values of their respective f-NP drugs, which means that these functionalized NP drugs are nontoxic to macrophages, and they show inhibition of the intramacrophage parasite at very low concentrations. Additionally, the mice *in vivo* toxicity parameters through hepatic and renal biochemical assays through the AST, ALT, creatine, and urea results have also confirmed the nontoxic nature of f-Comp-AmB, which has been in agreement with our previous studies with f-Grap-AmB (Mudavath et al., 2014) and f-CNT-AmB (Prajapati et al., 2011a,b).

In conclusion, the carbon-based f-Comp-AmB made from two different components does not correspond to a new compound; rather it displays a synergy between the best effects of the two. The composites are least toxic with better antileishmanial activity in comparison with classical AmB, f-CNT-AmB, and f-Grap-AmB. These preliminary *in vitro* and preclinical studies provide a stepping-stone for generating better treatment options for visceral leishmaniasis.

## Data Availability Statement

The raw data supporting the conclusions of this article will be made available by the authors, without undue reservation.

## Ethics Statement

The animal study was reviewed and approved by the Central Animal Ethics Committee (CAEC), Banaras Hindu University (CAEC number Dean/2014/CAEC/615).

## Author Contributions

MG, OPS, ONS, and SS: conceptualization of the project. MG, PM, and AV: data curation. MG, PM, and OPS: data analysis and validation. MG, PM, AV, VV, AK, GY, and SM: laboratory investigation. OPS, ONS, and SS: project administration. ONS and SS: funding resources. MG, PM, AV, and OPS: visualization. MRG, PM, AV, OPS, ONS, and SS: writing original draft. MG, PM, AV, VV, AK, GY, SM, OPS, ONS, and SS: writing—review and editing. All authors contributed to the article and approved the submitted version.

## Conflict of Interest

The authors declare that the research was conducted in the absence of any commercial or financial relationships that could be construed as a potential conflict of interest.

## Supplementary Material

Supplementary Figure 1 Giemsa-stained dabbed smears of *L. donovani*–infected hamster spleen tissue at 100X. White arrows indicate *L. donovani* amastigotes in untreated, AmB, and f-Comp-AmB groups.

Supplementary Figure 2 Spleen sizes of different experimental groups in hamsters.

Supplementary Table 1 *In vivo* biochemical parameters for hepatic and renal functions in Swiss albino mice. *n*, number of mice; ALT, Alanine aminotransferase; AST, Aspartate transaminase; AmB, Amphotericin B; f-CNT, Amine-modified carbon nanotubes; f-CNT-AmB, AmB conjugated with CNT; f-Grap, Amine-modified graphene; f-Grap-AmB, AmB conjugated with f-Grap; f-Comp, Amine-modified composite; f-Comp-AmB, AmB conjugated with f-composite. The results were represented as Mean ± *SD*.

Supplementary Table 2 *In vivo* efficacy studies in Syrian Golden hamsters using AmB, f-CNT, f-Grap, f-Comp, f-CNT-AmB, f-Grap-AmB, and f-Comp-AmB against experimental leishmaniasis. The results were analyzed using one-way ANOVA and expressed as Mean ± *SD* with *p* < 0.05 representing significance.
