# Supplementary figures and images for: Evaluation of Safety and Antileishmanial Efficacy of Amine Functionalized Carbon-Based Composite Nanoparticle Appended With Amphotericin B: An in vitro and Preclinical Study
Source: Front Chem. 2020 Jul 3;8:510. doi: 10.3389/fchem.2020.00510 (PMC7350933; doi:10.3389/fchem.2020.00510)

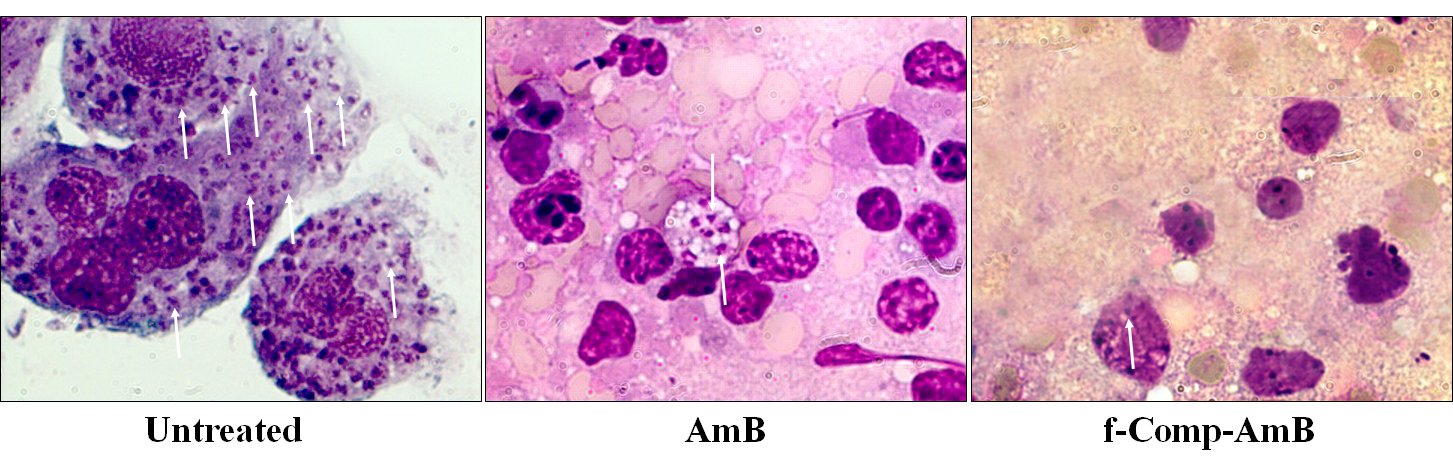

Supplement: Supplementary Figure 1 — Giemsa-stained dabbed smears of L. donovani–infected hamster spleen tissue at 100X. White arrows indicate L. donovani amastigotes in untreated, AmB, and f-Comp-AmB groups. [file Image_1.TIF]

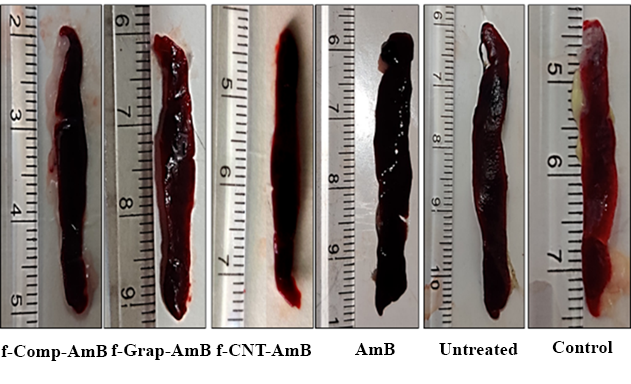

Supplement: Supplementary Figure 2 — Spleen sizes of different experimental groups in hamsters. [file Image_2.TIF]
