# Supplementary material for: Evaluation of Safety and Antileishmanial Efficacy of Amine Functionalized Carbon-Based Composite Nanoparticle Appended With Amphotericin B: An in vitro and Preclinical Study
Source: Front Chem. 2020 Jul 3;8:510. doi: 10.3389/fchem.2020.00510 (PMC7350933; doi:10.3389/fchem.2020.00510)
Supplement: Supplementary Table 1 — In vivo biochemical parameters for hepatic and renal functions in Swiss albino mice. n, number of mice; ALT, Alanine aminotransferase; AST, Aspartate transaminase; AmB, Amphotericin B; f-CNT, Amine-modified carbon nanotubes; f-CNT-AmB, AmB conjugated with CNT; f-Grap, Amine-modified graphene; f-Grap-AmB, AmB conjugated with f-Grap; f-Comp, Amine-modified composite; f-Comp-AmB, AmB conjugated with f-composite. The results were represented as Mean ± SD. [file Table_1.docx]

|  | **Control** | **Vehicle Control** | **AmB** | | | **f-Comp** | | | **f-Comp-AmB** | | |
| --- | --- | --- | --- | --- | --- | --- | --- | --- | --- | --- | --- |
|  | **5 mg/kg (n=4)** | **5 mg/kg (n=4)** | **5 mg/kg (n=4)** | **10 mg/kg (n=4)** | **20 mg/kg (n=4)** | **5 mg/kg (n=4)** | **10 mg/kg (n=4)** | **20 mg/kg (n=4)** | **5 mg/kg (n=4)** | **10 mg/kg (n=4)** | **20 mg/kg (n=4)** |
| **ALT (IU/L)** | 44.50±6.455 | 49.00±10.71 | 50.25±9.743 | 49.00±10.61 | 42.75±5.909 | 44.25±5.737 | 42.25±6.602 | 43.75±7.089 | 46.75±6.397 | 42.75±15.17 | 43.75±11.56 |
| **AST (IU/L)** | 44.25±6.131 | 47.00±9.899 | 47.00±12.96 | 44.75±11.95 | 44.75±7.932 | 45.50±8.851 | 44.25±7.274 | 43.00±6.325 | 51.00±5.944 | 45.50±7.047 | 43.25±4.787 |
| **Creatinine (mg/dl)** | 0.3750±0.1542 | 0.4250±0.1323 | 0.4850±0.07767 | 0.4250±0.1290 | 0.3900±0.09933 | 0.3700±0.1236 | 0.3450±0.1047 | 0.3925±0.1109 | 0.4125±0.1021 | 0.4175±0.08302 | 0.4350±0.04796 |
| **Urea (mg %)** | 15.75±3.594 | 17.25±3.096 | 17.50±4.041 | 18.75±2.062 | 16.25±3.775 | 16.25±3.500 | 14.75±2.500 | 15.75±3.594 | 16.75±3.304 | 16.25±4.992 | 16.75±3.862 |
|  | **10 mg/kg (n=4)** | **10 mg/kg (n=4)** |  |  |  | **f-Grap** | | | **f-Grap-AmB** | | |
| **ALT (IU/L)** | 41.25±11.30 | 41.25±12.09 |  |  |  | 43.75±8.261 | 43.50±13.03 | 41.75±9.946 | 51.25±5.123 | 47.50±10.66 | 44.75±9.430 |
| **AST (IU/L)** | 44.50±5.323 | 41.50±6.658 |  |  |  | 42.75±9.979 | 42.50±8.699 | 38.75±8.539 | 46.75±8.655 | 46.75±6.397 | 45.25±4.573 |
| **Creatinine (mg/dl)** | 0.3500±0.08907 | 0.4025±0.1350 |  |  |  | 0.3950±0.08021 | 0.3900±0.1080 | 0.3425±0.06850 | 0.3825±0.08694 | 0.4550±0.05916 | 0.4150±0.08386 |
| **Urea (mg %)** | 16.50±2.380 | 17.00±4.243 |  |  |  | 15.75±2.754 | 16.50±2.517 | 16.50±4.435 | 16.00±4.967 | 18.00±4.830 | 17.50±2.646 |
|  | **20 mg/kg (n=4)** | **20 mg/kg (n=4)** |  |  |  | **f-CNT** | | | **f-CNT-AmB** | | |
| **ALT (IU/L)** | 37.50±6.807 | 41.50±8.062 |  |  |  | 44.25±9.535 | 45.00±8.406 | 40.25±14.61 | 45.00±9.626 | 48.00±5.657 | 43.00±12.73 |
| **AST (IU/L)** | 41.25±8.382 | 43.25±6.602 |  |  |  | 45.75±7.890 | 45.75±9.777 | 35.75±7.890 | 50.50±9.983 | 50.50±10.63 | 41.75±11.59 |
| **Creatinine (mg/dl)** | 0.4000±0.07257 | 0.4275±0.09946 |  |  |  | 0.3525±0.1044 | 0.3225±0.09845 | 0.3225±0.08770 | 0.3875±0.05560 | 0.3975±0.07632 | 0.3725±0.03862 |
| **Urea (mg %)** | 16.00±3.916 | 16.75±4.031 |  |  |  | 15.75±4.856 | 17.75±2.500 | 15.00±4.082 | 16.25±3.500 | 18.75±6.551 | 18.25±2.500 |

**Supplementary Table 1.** *In vivo* biochemical parameters for hepatic and renal functions in Swiss albino mice. n, Number of mice; ALT, Alanine aminotransferase; AST, Aspartate transaminase; AmB, Amphotericin B; f-CNT, Amine-modified carbon nanotubes; f-CNT-AmB, AmB conjugated with CNT; f-Grap, Amine-modified graphene; f-Grap-AmB, AmB conjugated with f-Grap; f-Comp, Amine-modified composite; f-Comp-AmB, AmB conjugated with f-composite. The results were represented as Mean ± S.D

| **Group** | **Before treatment(n=8)** | **^b^f-CNT-AmB(n=24)** | **^c^f-Grap-AmB(n=24)** | **^d^f-Comp-AmB(n=24)** | **^a^****AmB(n=24)** | **f-CNT(n=24)** | **f-Grap(n=24)** | **f-Comp(n=24)** | **Control(n=8)** | **p value<;**  **^a vs b, c, d^** |
| --- | --- | --- | --- | --- | --- | --- | --- | --- | --- | --- |
| Parasites/500 nuclei Mean ± S.D | 3378 ±428.4 | 422.5 ±22.66 | 243 ±35.67 | 131.8 ±14.08 | 858.3± 97.66 | 2420 ±376.1 | 2464 ±364 | 2546 ±480.3 | 5974 ±217.7 | 0.001  0.001  0.001 |
| Spleen weight in grams, | 1.005±0.0203 | 0.8233±0.0906 | 0.7188±0.0516 | 0.6376±0.065 | 0.9308±0.0933 | 1.127±0.0698 | 1.19±0.103 | 1.114±0.0839 | 1.4±0.0944 | ns  0.05  0.01 |
| LDU × (10000) | 340.1±49.9 | 34.93±5.57 | 17.38±2.075 | 8.351±0.633 | 79.97±12.72 | 272.2±40.35 | 292±42.04 | 281.2±39.86 | 834.8±29.29 | 0.001  0.001  0.001 |
| % suppression of parasite replication |  | 92.92±0.4802 | 95.92±0.7118 | 97.79±0.2375 | 85.66±1.164 |  |  |  |  | 0.001  0.001  0.001 |
| % inhibition |  | 87.36±1.613 | 92.77±1.039 | 96.04±0.7526 | 74.13±5.338 |  |  |  |  | 0.01  0.001  0.001 |

**Supplementary Table 2.** *In vivo* efficacy studies in Syrian golden hamsters using AmB, f-CNT, f-Grap, f-Comp, f-CNT-AmB, f-Grap-AmB and f-Comp-AmB against experimental leishmaniasis. The results were analyzed using one way ANOVA and expressed as Mean ± S.D with p<0.05 represent significance.
